# Metabolic toxicity and neurological dysfunction in methylmalonic acidemia: from mechanisms to therapeutics

**DOI:** 10.1186/s10020-025-01395-z

**Published:** 2025-11-26

**Authors:** Mengmeng Du, Miaomiao Li, Shengnan Wu, Xue Wu, Yongxing Chen, Changlian Zhu

**Affiliations:** 1 Department of Endocrinology, Genetics and Metabolism, Children’s Hospital Affiliated to Zhengzhou University, Zhengzhou, China; 2 Henan Pediatric Clinical Research Center and Key Laboratory of Child Brain Injury, Institute of Neuroscience and Third Affiliated Hospital of Zhengzhou University, Zhengzhou, China; 3 Center for Brain Repair and Rehabilitation, Institute of Neuroscience and Physiology, University of Gothenburg, Göteborg, Sweden

**Keywords:** Methylmalonic academia, Toxic metabolites, Post-translational modification, Mitochondrial dysfunction, Neuroinflammation, Excitotoxicity, Therapeutic strategies

## Abstract

Methylmalonic acidemia (MMAemia) is an inborn error of organic acid metabolism characterized by the accumulation of toxic metabolites—including methylmalonic acid (MMA), 2-methylcitric acid (2-MCA), propionic acid (PA), homocysteine (Hcy), ammonia, and lactate—due to defects in methylmalonyl-CoA mutase or impaired cobalamin metabolism. These metabolites exert profound effects on the central nervous system, contributing to neurological injury through tightly interconnected mechanisms, including mitochondrial dysfunction, neuroinflammation, and excitotoxicity. This review synthesizes current evidence on how these metabolites trigger neurological dysfunction, integrating findings from clinical studies, animal models, and cellular systems. We also highlight the increasingly recognized role of aberrant post-translational modifications (e.g., methylmalonylation, propionylation, lactylation) in disrupting metabolic network architecture and reprogramming cellular metabolism. Despite advances in supportive therapies, intracerebral metabolite accumulation remains a therapeutic challenge. We discuss emerging strategies targeting mitochondrial protection, redox homeostasis, and inflammation—including enzyme replacement, gene therapy, antioxidant regimens, and exosome-based delivery. A deeper mechanistic understanding of metabolite-driven neurotoxicity is critical to the development of targeted interventions that can improve neurological outcomes in MMAemia.

## Introduction

**Fig. 1:**
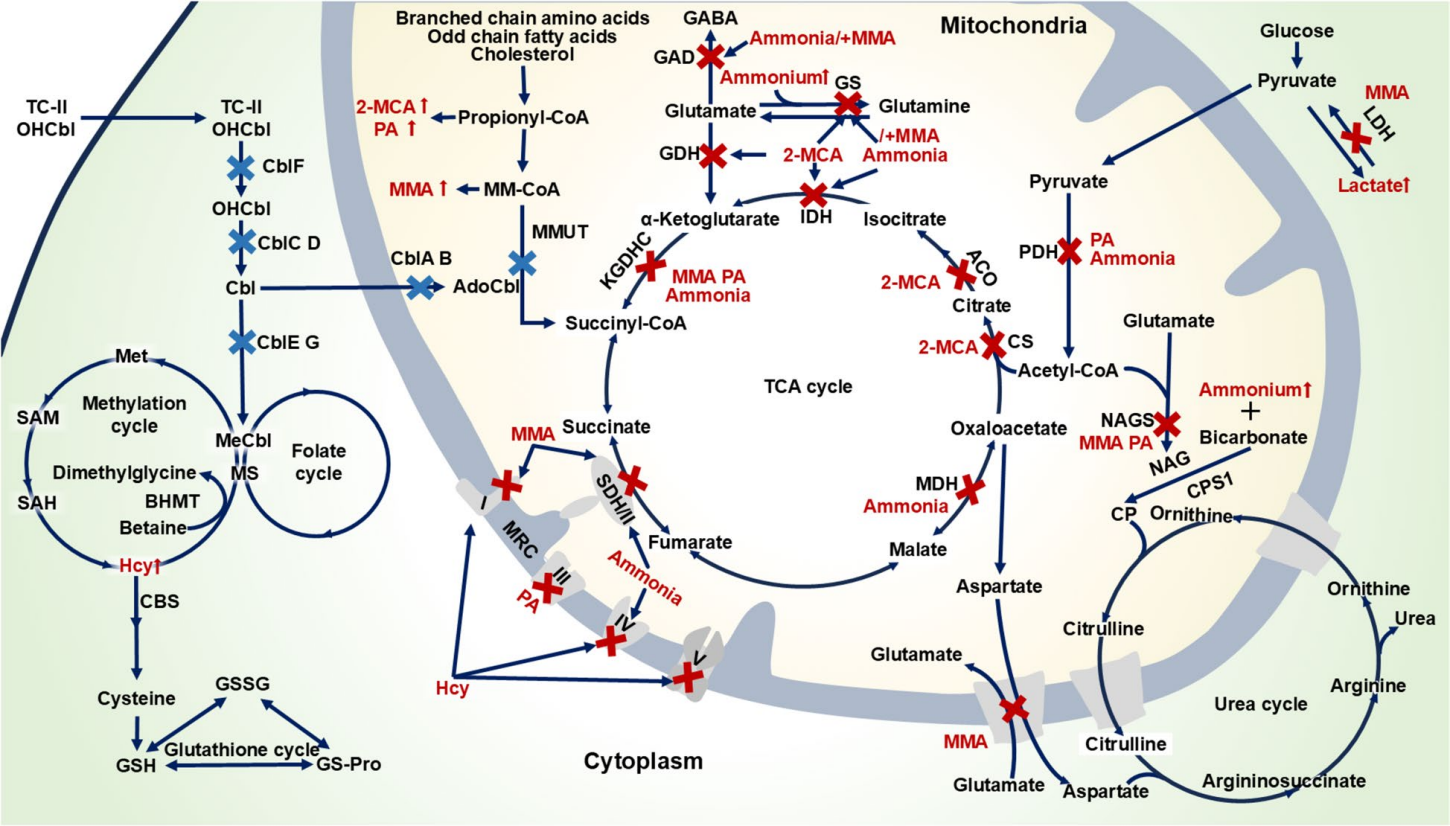
Dysregulated metabolic pathways and metabolite-mediated enzyme inhibition in MMAemia. Metabolic pathways include the propionate oxidation pathway, TCA cycle, MRC, urea cycle, glucose metabolism, and methylation and folate cycles. In the MMAemia schematic, accumulated metabolites are marked with red arrows, mutant pathways are denoted by blue crosses, and enzyme inhibition induced by metabolites (MMA, 2-MCA, PA, Hcy, ammonia and lactate) is indicated by red crosses. Abbreviations: ACO, aconitase; BHMT, betaine-homocysteine methyltransferase; CBS, cystathionine β-synthase; CS, citrate synthase; CP, carbamoyl phosphate; CPS1, carbamoyl phosphate synthetase 1; GSSG, oxidized glutathione; GS-Pro, glutathione–protein mixed disulfide; GLS, glutaminase; IDH, isocitrate dehydrogenase; Met, methionine; MM-CoA, methylmalonyl-CoA; SAM, S-adenosylmethionine; SAH, S-adenosylhomocysteine

Methylmalonic acidemia (MMAemia) is an inborn error of metabolism caused by defects in methylmalonyl-CoA mutase (MMUT) or cobalamin (Cbl) metabolism (Longo et al. 2022). Worldwide, its prevalence among newborns is 1.14 per 100,000, demonstrating an increasing trend over time, whereas the prevalence in clinically suspected patients reaches 652.11 per 100,000 (Jin et al. 2022). MMAemia is characterized by the pathological accumulation of metabolites, including methylmalonic acid (MMA), 2-methylcitric acid (2-MCA), propionic acid (PA), homocysteine (Hcy), ammonia and lactate (Ling et al. 2025) (Fig. 1). These metabolites are closely associated with brain damage, frequently manifesting as neurological dysfunction and structural brain abnormalities (Chen et al. 2023), yet the precise mechanisms underlying metabolite-induced neuropathology remain only partially understood.

The relationship between metabolites and brain damage in MMAemia has long been a focal point of intensive research. The high energy demand of the brain, the gate-keeping function of the blood–brain barrier (BBB), a high lipid content, vulnerable neuronal subpopulations, and glutamatergic neurotransmission all make the brain particularly vulnerable against mitochondrial dysfunction, neuroinflammation and excitotoxicity (Kölker et al. 2013). Although experimental and clinical studies demonstrate metabolite-mediated neurotoxicity in MMAemia (Denley et al. 2025; Li et al. 2021), critical knowledge gaps persist. First, insufficient integration of findings from biochemical studies, animal models, and clinical observations results in a fragmented understanding of injury pathways. Second, controversy persists regarding the dominant pathological mechanisms, specifically whether mitochondrial impairment or neuroinflammatory processes constitute the primary driver of neuronal injury. Third, the temporal progression of damage, particularly the transition from acute metabolic crises to chronic neurological sequelae, and the synergistic interactions between accumulating metabolites remain poorly characterized. Finally, the fragmented nature of existing studies on metabolite-induced brain damage hinders a unified mechanistic understanding and limits the development of interventions to mitigate neurodevelopmental impairments.

These unresolved issues impede the translation of research findings into targeted clinically effective strategies. Therefore, a systematic review is essential to consolidate knowledge, resolve inconsistencies, and clarify the multifactorial interplay between metabolites and brain injury in MMAemia. By systematically integrating data from heterogeneous studies (spanning in vitro models, animal studies, and clinical cohorts), this review aims to: (1) integrate current evidence on the direct and indirect neurotoxic mechanisms mediated by MMA, 2-MCA, PA, Hcy, ammonia and lactate; (2) summarize the involvement of mitochondrial dysfunction, neuroinflammation, and neuroexcitotoxicity in the pathogenesis of MMAemia; (3) synthesize therapeutic strategies modulating metabolic pathways and mechanisms. By synthesizing existing evidence on potential associations between metabolites and neuropathology, this integrated analysis seeks to offer a preliminary framework that may help prioritize candidate therapeutic targets, provide clues for personalized intervention strategies, and inform future research aimed at improving neurological outcomes in MMAemia.

## Molecular mechanisms of metabolite-induced neurotoxicity

### Primary metabolites

MMAemia is an inborn error of metabolism caused by deficiency of MMUT and its cofactor Cbl, which are essential for branched-chain amino acid, odd-chain fatty acid, and cholesterol catabolism (Hu and Kong 2022). This defect leads to pathological accumulation of primary organic acids, including MMA, 2-MCA, and PA. Impaired Cbl metabolism disrupts the synthesis of adenosylcobalamin (AdoCbl) and methylcobalamin (MeCbl), the respective cofactors for MMUT and methionine synthase, resulting in Hcy accumulation alongside the characteristic organic acid (Lee and Kim 2022; Watkins and Rosenblatt 2022) (Fig. 1). The metabolites disrupt cellular homeostasis through synergistic mechanisms involving impaired energy metabolism, oxidative stress, neuroinflammation, excitotoxicity, and epigenetic dysregulation, culminating in irreversible neurological damage (Kölker et al. 2013; Head et al. 2023; Dimitrov et al. 2021).

#### MMA

The guideline panel recommended MMA, a metabolic byproduct of the propionate oxidation pathway, as the primary biomarker of MMAemia in plasma and urine (Forny et al. 2021; Baumgartner et al. 2014). Clinically, elevated MMA concentrations are commonly observed in plasma, cerebrospinal fluid, and urine. In severe cases, plasma and cerebrospinal fluid levels can reach approximately 2.5–5.0 mmol/L, while urinary excretion may exceed the reference range by several 100-fold (Zhou et al. 2024; Liu et al. 2024). Notably, central nervous system (CNS) concentrations often surpass peripheral levels, reflecting impaired efflux of brain-derived MMA across the BBB (Sauer et al. 2010). The neuropathological manifestations of MMAemia stem from the accumulation of MMA in the CNS (Lee and Kim 2022; Dimitrov et al. 2021). Even the most effective treatment currently available to improve metabolism-liver transplantation, which corrects peripheral metabolic deficiencies by restoring hepatic enzymatic activity, fails to mitigate the persistent intracerebral accumulation of MMA and associated neurotoxicity (Martinelli et al. 2023; Vernon et al. 2014; Kaplan et al. 2006). The mechanisms underlying MMA-induced brain damage are multifactorial, encompassing mitochondrial dysfunction (energy failure/oxidative stress), neuroinflammatory responses, excitotoxicity secondary to neurotransmitter imbalance and ultimately, cell death pathways.

##### Cognitive and behavioral consequences

As a neurotoxin, MMA induces neuronal injury and cognitive dysfunction through intracerebral accumulation. Clinically, this manifests as an inverse correlation between circulating MMA levels (particularly greater variability) and global cognitive scores (Ling et al. 2025). Notably, MMA in serum neuro-exosomes demonstrates superior diagnostic accuracy for cognitive impairment in patients with MMAemia (Sun et al. 2022). Beyond inherited disorders, elevated MMA levels are associated with accelerated cognitive decline (particularly in language processing) in aging populations and neuropathy in acquired conditions (Tejero et al. 2024; Mccracken et al. 2006; Sun et al. 2014). Animal models confirm that MMA induces specific neurobehavioral deficits (e.g., seizures, memory impairment), while sparing anxiety, locomotion, and reference memory (Gabbi et al. 2017; Fernandes et al. 2011; Zhou et al. 2024; Marisco et al. 2003; Vasques et al. 2006; Pettenuzzo et al. 2003; Ribeiro et al. 2013).

##### Energy failure and oxidative stress

Studies demonstrate that MMA impairs energy metabolism by inhibiting both glucose oxidation and ketone body utilization (Kolker and Okun 2005; Wajner and Coelho 1997). Specifically, it disrupts key metabolic pathways, including the tricarboxylic acid (TCA) cycle (via inhibition of α-ketoglutarate dehydrogenase complex [KGDHC] and succinate dehydrogenase [SDH], the latter also functioning as mitochondrial respiratory chain [MRC] complex II), the mitochondrial malate shuttle, pyruvate carboxylase, and β-hydroxybutyrate metabolism (Kolker and Okun 2005; Brusque et al. 2002; Jafari et al. 2013). In SH-SY5Y cells, it induces coenzyme Q10 deficiency and suppresses MRC complexes II-III activity (Proctor et al. 2020). Furthermore, MMA exhibits tissue-specific inhibition of MRC complexes (I, I-III, and II-III) (Brusque et al. 2002; Pettenuzzo et al. 2006). These metabolic disturbances lead to energy failure and exacerbate oxidative stress. MMA acts as a potent oxidant, elevating reactive oxygen species (ROS)/reactive nitrogen species (RNS) levels and causing mitochondrial permeability transition, outer membrane rupture and subsequent dysfunction (Tejero et al. 2024; Schuck et al. 2013; Melo et al. 2011; Colín-González et al. 2015; Klemmensen et al. 2024). ROS-mediated damage includes protein oxidation, lipid peroxidation, DNA lesions, and depletion of antioxidant defenses, collectively contributing to cell death (Fernandes et al. 2011; Pettenuzzo et al. 2003; Schuck et al. 2013; Colín-González et al. 2015; Fontella et al. 2000; Andrade et al. 2014; Viegas et al. 2014; Almeida et al. 2022).

##### Neuroinflammation and glial activation

MMA-induced neuroinflammation has been consistently demonstrated in preclinical and clinical studies, characterized by elevated interleukin (IL) family cytokines (IL-1β, IL-6), tumor necrosis factor-α (TNF-α), and significant glial activation (microglia and astrocytes) (Li et al. 2021, 2017; Gabbi et al. 2017; Ribeiro et al. 2013; Dos Reis et al. 2024). Activated microglia further secrete some cytokines, chemotactic factors, reactive oxidative species, NO, exacerbating CNS damage (Ribeiro et al. 2013; Li et al. 2017). MMA-mediated inhibition of the mitochondrial dicarboxylate carrier (Mirandola et al. 2008) impairs cellular function in a dual manner: it reduces mitochondrial succinate availability, leading to energy failure, while concurrently increasing extracellular succinate levels. Moreover, MMA acts as an agonist of the succinate receptor (Geubelle et al. 2017). Through this receptor activation, MMA may promote neuroinflammation by initiating GPCR-dependent signaling cascades (e.g., MAPK/ERK, PKA, PKC) and subsequently activating pro-inflammatory transcription factors such as NF-κB and p38 (Tejero et al. 2024; Gilissen et al. 2016). Collectively, these findings underscore MMA-driven neuroinflammation as a key contributor to neurological dysfunction.

##### Excitotoxicity and neurotransmitter disruption

MMA disrupts glutamate metabolism, causing N-methyl-D-aspartate (NMDA) receptor overactivation and concurrent inhibition of glutamate decarboxylase (GAD), which suppresses γ-aminobutyric acid (GABA) synthesis. This imbalance between excitatory and inhibitory neurotransmission precipitates excitotoxicity (Brusque et al. 2001; De Mattos-Dutra et al. 2000). Additionally, MMA alters acetylcholinesterase activity, exacerbating synaptic dysfunction (Affonso et al. 2013) Combined with MMA-induced myelin damage (Gabbi et al. 2017), these excitotoxicity-driven mechanisms ultimately lead to cognitive decline, epilepsy, and neurological deficits.

##### Neuronal death

MMA accumulation triggers neuronal death as the terminal effector of brain injury (Denley et al. 2025; Gabbi et al. 2017; Zhou et al. 2024; Luciani et al. 2021). Dose-dependent neuronal death (> 90% mortality) was observed in fetal rat striatal and cortical cultures following 24-h MMA exposure (Mclaughlin et al. 1998). MMA triggers time- and concentration-dependent apoptosis, likely through MAPK and p53 pathway dysregulation (Zhou et al. 2024; Han et al. 2015). Mechanistically, MMA reduces intracellular ATP/ADP ratios, inducing both ROS overproduction and mitochondrial membrane depolarization, which disrupts Bcl-2 family protein equilibrium (Mclaughlin et al. 1998; Li et al. 2014). This is evidenced by decreased Bcl-2 expression and increased Bax activation, promoting Bax mitochondrial translocation (Zhou et al. 2024). Subsequent cytochrome c release activates caspase-dependent apoptosis through dual pathways: 1) The intrinsic pathway via mitochondrial apoptogen release, and 2) The extrinsic pathway initiated by caspase-8 (activated by TNF-α/IL-1β-mediated caspase-1) which cleaves Bcl-2 proteins to amplify mitochondrial dysfunction (Gabbi et al. 2017; Van Opdenbosch and Lamkanfi 2019). Oxidative stress and cytochrome c release further potentiate caspase-3 activation, a key executor protease that also regulates neural development (Li et al. 2014; Asadi et al. 2022). Consequently, MMA-induced neuronal death arises from a self-amplifying cycle. Energy depletion contributes to mitochondrial dysfunction, leading to cytochrome c release and activation of the intrinsic apoptotic pathway. Simultaneously, this mitochondrial stress promotes neuroinflammation, which exacerbates damage through extrinsic apoptotic signaling—further amplifying caspase-3 activation and neuronal loss.

In summary, cerebral MMA retention disrupts mitochondrial energy homeostasis, exacerbates oxidative stress, and triggers neuroinflammation and excitotoxicity, collectively driving neuronal death and CNS dysfunction.

#### 2-MCA and PA

MMAemia is characterized by prominent accumulation of MMA and its precursor methylmalonyl-CoA, with additional pathological contributions from propionyl-CoA-derived metabolites, particularly 2-MCA and PA(Molema et al. 2018).

##### Mitochondrial damage and ammonia-mediated apoptosis caused by 2-MCA

2-MCA is a tricarboxylic acid derived from the condensation of propionyl-CoA and oxaloacetate (Maines et al. 2020). Although 2-MCA is present at lower concentrations than MMA, its levels are still elevated in patients with MMAemia. In dried blood spots, the median concentration of 2-MCA is 0.47 μmol/L in combined MMAemia and 1.81 μmol/L in isolated MMAemia—both exceeding the healthy reference range (0.02–0.27 μmol/L), yet markedly lower than the corresponding MMA concentrations (Wajner and Coelho 1997; Liu et al. 2024). Notably, 2-MCA levels correlate with disease burden and other metabolic biomarkers, including ammonia, glycine, lysine, C3, C3/C2, and C3/C16 (Maines et al. 2020). Moreover, 2-MCA may act synergistically with MMA in promoting neurotoxicity (Cudre-Cung et al. 2016). 2-MCA inhibits several critical enzymes of the TCA cycle, including citrate synthase, aconitase, and isocitrate dehydrogenase, thereby impairing mitochondrial energy homeostasis (Kölker et al. 2013). Additionally, 2-MCA inhibits glutamate dehydrogenase (GDH), reducing the conversion of glutamate to α-ketoglutarate and thus restricting TCA cycle flux (Amaral et al. 2016). Beyond metabolic interference, 2-MCA induces mitochondrial dysfunction by opening permeability transition pores, depolarizing membrane potential, and promoting swelling, mechanisms particularly detrimental to energy-demanding neural cells (Amaral et al. 2016). Low-dose 2-MCA in 3D rat brain cultures impairs axonal growth, causes astrocytic dysfunction, disrupts oligodendrocyte maturation, and induces glial apoptosis, evidenced by reduced p-NFM, decreased fibrous density, lower MBP expression, and caspase-3 activation respectively (Jafari et al. 2013). Further mechanistic studies uncovered that 2-MCA exposure induces elevated ammonium levels concomitant with decreased glutamine in culture media, an early event preceding cellular degeneration and apoptosis (Cudre-Cung et al. 2016). The observed neurotoxicity may be mediated by the 2-MCA-induced inhibition of glutamine synthetase (GS) activity and the disruption of glutamine metabolism, ultimately leading to ammonia-mediated cerebral toxicity (Cudre-Cung et al. 2016).

##### Neurobehavioral deficits and multimodal toxicity induced by PA

PA is a key pathological metabolite shared by MMAemia and propionic acidemia (Kölker et al. 2013). Clinically, PA accumulation is reflected by elevated C3 and an increased C3/C2 ratio in dried blood spots, along with increased urinary excretion of 3-hydroxypropionic acid. Despite distinct enzymatic defects (MMUT versus propionyl-CoA carboxylase deficiency), both disorders exhibit neurological sequelae, including seizures, developmental delay, and structural brain abnormalities (Ballhausen et al. 2009). The neurotoxic effects of PA are mediated through multiple mechanisms. While PA consistently induces autism-like behaviors across studies, its effects on learning and memory remain controversial—with some reporting deficiencies (El-Ansary et al. 2012; Erten 2021) and others no significant impact (Lobzhanidze et al. 2020). These discrepancies may arise from administration route-dependent neurotoxicity: intrastriatal PA triggers NMDA receptor-mediated excitotoxicity and seizures (Rigo et al. 2006), whereas systemic delivery inhibits GABA transaminase, causing neuronal suppression and lethargy (Morland et al. 2018). Differential cerebral PA concentrations resulting from distinct administration routes likely underlie these varied outcomes. Compared to MMA, PA exhibits more potent lipid biosynthesis inhibition (De Mello et al. 1997). Accordingly, PA exposure may induce synaptic dysfunction through reduced hippocampal dendritic spine density, contributing to rodent autism-like behaviors (Lobzhanidze et al. 2020). Additional mechanisms for these behavioral manifestations may involve PA-induced dendritic spine loss and impaired synaptic plasticity (Choi et al. 2020). Furthermore, PA disrupts cytoskeletal integrity by rearranging actin, vimentin, and glial fibrillary acidic protein, impairing the normal structure and function of neurons and glial cells (De Mattos-Dutra et al. 2000; De Almeida et al. 2006). Collectively, PA disrupts brain function through neurotransmitter imbalance (GABA/NMDA dysregulation), synaptic dysfunction, and cytoskeletal destabilization.

##### Mitochondrial dysfunction induced by PA

Energy failure and oxidative stress are key pathological hallmarks of PA-mediated neurotoxicity in propionic acidemia and MMAemia (Sidorina et al. 2024). PA and propionyl-CoA exert profound neurotoxicity by disrupting cellular energy metabolism and mitochondrial function, primarily through broad-spectrum inhibition of key metabolic enzymes, including pyruvate dehydrogenase (PDH) complex, KGDHC, succinyl-CoA ligase, N-acetylglutamate synthase (NAGS), Na^+^,K^+^-ATPase, and MRC complex III (Kölker et al. 2013; Morath et al. 2008; Wyse et al. 1998). These inhibitory effects impair ATP production, urea cycle function, and mitochondrial bioenergetics, exacerbating systemic metabolic dysfunction. PA preferentially targets GABAergic neurons and glial cells, disrupting their energy metabolism and exhibiting selective striatal vulnerability (where GABAergic neurons comprise > 90% of neurons), while glial metabolic impairment further compromises intercellular substrate shuttling (Morland et al. 2018; Nguyen et al. 2007). PA elevates ROS, causing lipid peroxidation, protein carbonylation, and oxidative damage in the rat cerebral cortex, which disrupts membrane integrity and oxidizes critical biomolecules (Fontella et al. 2000; Rigo et al. 2006). PA further disrupts autophagic flux via MAPK/ERK signaling activation, resulting in impaired organelle degradation and insufficient ROS elimination (Choi et al. 2020). Moreover, PA impairs key components of the glutathione redox system (e.g., glutathione peroxidase [GPx] and reduced glutathione [GSH]) and other antioxidant enzymes, including superoxide dismutase (SOD) and catalase (CAT) (Fernandes et al. 2011; El-Ansary et al. 2012; Khalil et al. 2015). Due to the high metabolic demand and oxidative vulnerability of the brain, sustained ROS exposure triggers neuronal death and functional impairment (Kölker et al. 2013).

##### Neuroinflammation induced by PA

PA triggers neuroinflammation, as demonstrated by increased levels of pro-inflammatory cytokines (e.g., IL-1α, IL-6, IL-8, and TNF-α) in the brain (El-Ansary et al. 2012; Erten 2021). Mechanistically, PA inhibits the Nrf2/HO-1 pathway while activating NF-κB, generating a pro-oxidant and pro-inflammatory environment that disrupts apoptotic protein balance and promotes neuronal death (El-Ansary et al. 2012; Erten 2021; Khalil et al. 2015). Collectively, PA exerts neurotoxicity through multiple pathways involving metabolic impairment, oxidative stress, neuroinflammation, and structural–functional neuronal damage.

In summary, accumulated organic acids act in concert to induce irreversible neurological damage in MMAemia through synergistic mechanisms involving impaired energy metabolism, oxidative stress, neuroinflammation, and excitotoxicity.

#### Hcy

Hcy, a sulfur-containing amino acid derived from methionine metabolism, has a normal fasting plasma concentration of < 15 μmol/L (Olivieri et al. 2025). Clinically, serum Hcy levels are used to classify MMA into two subtypes for therapeutic decision-making: isolated MMAemia (without hyperhomocysteinemia) and combined MMAemia with hyperhomocysteinemia (MMAemia-HC, elevated Hcy and MMA) (Hu and Kong 2022; Haijes et al. 2019).

##### Disrupted methylation and metabolic imbalance

Three interdependent pathways regulate Hcy metabolism (Korczowska-Lacka et al. 2023). (1) In the methylation cycle, Hcy undergoes remethylation to methionine catalyzed by methionine synthase (MeCbl-dependent) or betaine-homocysteine methyltransferase. In MMAemia-HC, impaired MeCbl metabolism compromises methionine synthase, leading to Hcy accumulation and methionine depletion. This dual perturbation severely impairs cellular methylation capacity: elevated Hcy increases S-adenosylhomocysteine (a potent methyltransferase inhibitor), while methionine deficiency limits the synthesis of S-adenosylmethionine, the universal methyl donor (Lee and Kim 2022; Saija et al. 2025). Such methylation dysfunction may induce epigenetic alterations, as demonstrated by CpG hypomethylation in the presenilin gene promoter during hyperhomocysteinemia (Fuso et al. 2011). (2) The transsulfuration pathway diverts Hcy to cystathionine through cystathionine β-synthase-mediated conversion, generating excretable sulfur compounds and supporting glutathione synthesis—a critical link between Hcy metabolism and redox homeostasis (Pastore et al. 2014). (3) Under pathological conditions, accumulated Hcy diffuses passively into circulation, with the majority binding covalently to albumin and the remainder circulating freely. Although albumin shows limited BBB permeability (Smith and Refsum 2016), elevated Hcy can disrupt this compartmentalization. Experimental evidence from rodent models reveals that vitamin B-deficient (including B12-deficient) offspring exhibit twofold higher plasma Hcy levels, accompanied by a 34% increased brain Hcy accumulation—particularly in the cerebellum, hippocampus, striatum, and subventricular zone (Blaise et al. 2007), consistent with clinical observations linking maternal B12 deficiency to secondary (non-genetic) neonatal MMAemia-associated neurological deficits (Pajares et al. 2021).

##### Neurobehavioral deficits

Substantial evidence connects Hcy levels with cognitive performance spanning multiple domains (including memory, executive function, visual processing, language, reasoning, information processing, and attention) in healthy individuals (Luzzi et al. 2022; Cebi et al. 2022; Agrawal et al. 2015; Raszewski et al. 2015). Clinical studies consistently associate elevated Hcy with cerebrovascular pathology (cerebral atherosclerosis, ischemic stroke and lacunar infarction), cognitive impairment (dementia and Alzheimer's disease), white matter lesions, and brain atrophy, and establish Hcy as an independent risk factor for multiple CNS disorders (Luzzi et al. 2022; Pajares et al. 2021; Ji et al. 2019; Lee et al. 2017; Maesato et al. 2017). Rodent studies corroborate these findings, showing Hcy-induced seizures, anxiety-like behaviors, cognitive deficits, motor dysfunction, and hippocampal atrophy (Luzzi et al. 2022; Djuric et al. 2018; Troen et al. 2008; Folbergrova et al. 2010; Wyse et al. 2020).

##### Mitochondrial dysfunction and neuroinflammation

Hcy disrupts neurological function through interconnected mechanisms involving mitochondrial impairment, oxidative stress, and neuroinflammation. By inhibiting MRC complex (e.g., I, II, IV and V) and Na^+^,K^+^-ATPase activity, Hcy impairs bioenergetics while concurrently inducing oxidative damage through dysregulation of antioxidant enzymes (e.g., SOD, CAT and GPx) (Longoni et al. 2018; Kolling et al. 2016; Cordaro et al. 2021). Beyond its mitochondrial toxicity, Hcy exacerbates neuroinflammation through multiple synergistic pathways. It activates NF-κB transcriptional activity while suppressing HO-1 expression, driving astrocytic reactivity and production of pro-inflammatory mediators including cytokines (TNF-α, IL-1β and IL-6), chemokines, and prostaglandin E2 (Djuric et al. 2018; Longoni et al. 2018). Hcy triggers microglial activation, promoting pyroptosis via the NLRP3/caspase-1/GSDMD axis and ferroptosis through SIRT1/SLC7A11 pathway (Yin et al. 2025; Yang et al. 2024b). These processes are characterized by upregulated pro-inflammatory mediators (e.g., IL-1β, IL-6 and TNF-α), suppressed anti-inflammatory cytokines (e.g., IL-4 and IL-10) and iron dysregulation (Fe^2^⁺ accumulation). The convergence of bioenergetic failure, oxidative stress, and neuroinflammatory responses drives Hcy-mediated neurotoxicity.

##### Excitotoxicity and neurotransmitter imbalance

Hcy enhances glutamatergic neurotransmission by synergistically activating both NMDA receptors and group I metabotropic glutamate receptors (Djuric et al. 2018; Zieminska et al. 2003). Functioning as an endogenous NMDA receptor agonist, Hcy activates GluN2A subunits at low concentrations (< 100 μmol/L), inducing calcium overload. At higher concentrations (> 200 μmol/L), its thiol-mediated reduction of receptor disulfide bonds preferentially enhances GluN2B-containing receptor activity, leading to exacerbated calcium influx and progressive excitotoxic neuronal damage (Sibarov et al. 2020). The calcium overload activates calcium-dependent enzymes (e.g., calpain, nitric oxide synthase [NOS]), generating ROS and mitochondrial dysfunction, which triggers the release of apoptosis-inducing factor and cytochrome C, thereby activating caspase-9 and caspase-3. Furthermore, Hcy upregulates pro-apoptotic proteins (p53, Bax) and downregulates Bcl-2, exacerbating neuronal apoptosis. The ERK/p38 MAPK signaling pathway may also be involved in this process (Ivanova et al. 2020).

##### Vascular pathology and BBB dysfunction

Hcy disrupts vascular function through multiple pathogenic mechanisms. By inhibiting endothelial NOS, Hcy reduces nitric oxide (NO) bioavailability, thereby impairing endothelium-dependent vasodilation (Lai and Kan 2015). Concurrently, it induces vascular inflammation and oxidative stress, triggering endothelial cell death (including apoptosis, pyroptosis, and ferroptosis) while promoting vascular smooth muscle cell proliferation and extracellular matrix (ECM) remodeling (Lai and Kan 2015; Ortiz-Salguero et al. 2024). These cumulative effects disrupt structural integrity and increase vascular stiffness, ultimately enhancing vascular permeability. Furthermore, Hcy enhances thrombosis risk through procoagulant microparticle-mediated platelet activation and antithrombin III inhibition, thereby predisposing to microvascular infarction (Hainsworth et al. 2016). The BBB, a highly specialized vascular structure, exhibits marked susceptibility to Hcy-mediated injury by inducing MMP-9 activation, which has been shown to degrade tight junction proteins, particularly claudin-5 and occludin (Zhong et al. 2019; Tawfik et al. 2021). Astrocyte-endothelial interaction disruption within the neurovascular unit exacerbates barrier dysfunction, enabling blood-derived neurotoxins to infiltrate the parenchyma and induce tissue damage. Additionally, the osmotic gradient imbalance between the vascular and ventricular systems drives pathological water influx into the ventricles, potentially culminating in hydrocephalus (Ortiz-Salguero et al. 2024).

In summary, Hcy mediates neurotoxicity through interconnected pathways including mitochondrial dysfunction, neuroinflammatory activation, excitotoxicity, vascular/BBB disruption, and methylation abnormalities, collectively exacerbating brain injury in MMAemia-HC.

### Secondary metabolites

During metabolic decompensation in MMAemia, the accumulation of primary metabolites disrupts biochemical pathways, resulting in excessive production of secondary toxic metabolites, including ammonia and lactic acid (Longo et al. 2022; Ribas et al. 2022). Methylmalonyl-CoA and propionyl-CoA competitively inhibit NAGS, reducing NAGS activity by over 30% and consequently decreasing N-acetylglutamate (NAG) production. As NAG serves as an essential allosteric activator of carbamoyl phosphate synthetase I, this inhibition impairs ammonia clearance, culminating in hyperammonemia (Lee and Kim 2022). Furthermore, 2-MCA directly inhibits GS activity, disrupting the conversion of glutamate and ammonium to glutamine and thereby exacerbating ammonia accumulation. This pathological process is further amplified through a vicious cycle wherein excess ammonia induces inducible NOS (iNOS)-mediated GS nitration (Cudre-Cung et al. 2016). Concurrently, elevated MMA promotes lactate accumulation through two distinct mechanisms. First, it competitively inhibits lactate dehydrogenase (LDH), thereby reducing lactate clearance. Second, it induces mitochondrial dysfunction, which impairs pyruvate oxidation and further contributes to lactate buildup (Tejero et al. 2024) (Fig. 1).

#### Ammonia

##### Ammonia homeostasis disruption

Endogenous ammonia production arises from at least 16 enzymatic pathways (Felipo and Butterworth 2002), with three predominant sources: (1) glutaminase-mediated glutamine hydrolysis, (2) GDH-catalyzed oxidative deamination of glutamate, and (3) the purine nucleotide cycle (Cudre-Cung et al. 2016; Rothman et al. 2012). Ammonia detoxification primarily depends on the urea cycle (Head et al. 2023), with its impairment in MMAemia explaining why approximately 70% of patients develop hyperammonemia (Gabbi et al. 2018). Although the brain lacks a complete urea cycle, physiological ammonia levels are maintained through slower amino acid catabolism than in the liver and efficient astrocytic glutamine synthesis (Sen et al. 2025). In MMAemia, however, GS suppression disrupts nitrogen homeostasis by impairing detoxification pathways (Jafari et al. 2013). The metabolic disturbance markedly increases neural susceptibility to ammonia toxicity due to elevated metabolic requirements and constrained detoxification capacity.

##### Neurobehavioral deficits and mechanisms

Both acute and chronic hyperammonemia induce irreversible neurological damage (Oja et al. 2017), with particularly severe consequences for the developing brain (Ribas et al. 2022; Gabbi et al. 2018). The characteristic neuropathology includes cerebral edema, atrophy, white matter abnormalities, and ventricular enlargement, progressing to seizures, coma, and cognitive impairment-clinical manifestations consistently exacerbated by concurrent acidosis or systemic inflammation (Longo et al. 2022; Oja et al. 2017; Shawcross et al. 2004; Bachmann 2002). Rodent studies of MMA-related hyperammonemia reveal selective neurobehavioral effects, inducing working memory deficits and seizures while sparing motor function, reference memory, and anxiety-like behaviors (Gabbi et al. 2018; Royes et al. 2016). Mechanistically, these effects involve interconnected pathways: mitochondrial dysfunction, neuroinflammation, disrupted glutamate-glutamine cycling, osmotic imbalance, impaired glutamatergic signaling, and dysregulated cerebrovascular autoregulation (Oja et al. 2017; Bjerring et al. 2009).

##### Mitochondrial dysfunction

Ammonia-induced mitochondrial dysfunction represents a pivotal mechanism contributing to neuropathology in MMAemia. Ammonia directly inhibits critical TCA cycle enzymes, including KGDH, isocitrate dehydrogenase, and malate dehydrogenase, compromising oxidative phosphorylation (OXPHOS) (Prasad et al. 2024). It also impairs MRC complexes (e.g., II and IV), further reducing ATP synthesis (Heidari 2019). PDH inhibition by ammonia exacerbates energy depletion by limiting TCA cycle substrates and promoting lactate accumulation (Ribas et al. 2022; Sorensen et al. 2024). Overall, ammonia suppresses mitochondrial energy production. Ammonia markedly elevates ROS, RNS and NO, inducing protein tyrosine nitration, lipid peroxidation, S-nitrosylation of cysteine residues, and nucleic acid oxidation (Redolfi-Bristol et al. 2024; Angelova et al. 2022; Skowronska and Albrecht 2013). It concurrently compromises antioxidant defenses by suppressing SOD, CAT, GPx, and glutathione synthesis, thereby exacerbating oxidative stress (Heidari 2019). Through the glutamine-mediated "Trojan Horse" mechanism, ammonia accumulates in mitochondria, inducing mitochondrial permeability transition pore opening, mitochondrial swelling, and cytochrome c release, ultimately triggering mitochondrial dysfunction and apoptosis (Zielinska et al. 2022).

##### Neuroinflammation and glial activation

Ammonia induces excessive activation of microglia and astrocytes, stimulating pro-inflammatory cytokine release (Claeys et al. 2025; Jo et al. 2021). Ammonia-mediated astrocyte activation exacerbates brain injury in MMAemia, as demonstrated by elevated glial fibrillary acidic protein expression in cortical, hippocampal, and striatal regions, along with increased TNF-α and IL-1β levels in murine models receiving combined ammonia and MMA treatment (Gabbi et al. 2018). In ammonia-exposed astrocytes, inflammatory markers including TNF-α, IL-1β, IL-6, iNOS, high-mobility group box 1, and cyclooxygenase-2 are significantly upregulated. Mechanistically, ammonia activates p38 MAPK and NF-κB pathways, triggering inflammatory cascades (Bobermin et al. 2020). The brain-specific *Mut*^−/−^ mouse model reveals that elevated ammonia and CCL11 levels in early disease stages recruit and activate microglia, contributing to the initiation of neuroinflammatory responses (Remacle et al. 2018). Inflammatory cytokines (e.g., TNF-α, IL-6 and IL-17) synergize with ammonia to disrupt BBB integrity, promoting CNS accumulation that induces astrocyte dysfunction and microglial activation, ultimately exacerbating neuronal damage (Sen et al. 2025; Claeys et al. 2025).

##### Glutamate/GABA-glutamine cycle and astrocytic

The glutamate/GABA-glutamine cycle plays an essential role in maintaining neurotransmission through astrocyte-mediated regulation (Andersen and Schousboe 2023). Within this cycle, astrocytes uptake synaptic glutamate and GABA, enzymatically converting glutamate and ammonia to glutamine via GS. Following intercellular transfer, neurons metabolize the astrocyte-derived glutamine to replenish both glutamate and GABA pools, thereby sustaining neurotransmitter homeostasis and excitatory-inhibitory balance (Andersen 2025). Ammonium chloride alone or in combination with MMA inhibits key metabolic enzymes GS and GAD, resulting in elevated synaptic glutamate levels and impaired GABAergic neurotransmission (Royes et al. 2016). These disturbances induce NMDA, α-amino-3-hydroxy-5-methyl-4-isoxazolepropionic acid (AMPA), and metabotropic glutamate (mGlu) receptor hyperactivation and synaptic dysfunction (Oja et al. 2017; Braissant et al. 2013). Beyond these specific mechanisms, hyperammonemia induces widespread neurotransmitter dysregulation, affecting serotonergic, cholinergic, dopaminergic and noradrenergic pathways, ultimately leading to complex neurotransmitter system dysregulation (Lee and Kim 2022; Oja et al. 2017). Astrocyte-derived glutamine functions as a vital osmolyte in brain volume regulation (Sen et al. 2025) Paradoxically, 2-MCA-treated 3D rat brain organoids demonstrate increased intracellular glutamine despite GS inhibition, concurrent with extracellular depletion, suggesting either transport impairment or compensatory mitochondrial glutaminogenesis (Cudre-Cung et al. 2016). While this maldistribution promotes astrocyte swelling, alternative evidence identifies the initial pathology as arising from disrupted astrocytic potassium buffering (Eid and Lee 2013). Specifically, ammonia competes with potassium uptake, elevating extracellular K⁺ levels that pathologically activate Na^+^-K^+^−2Cl^−^ cotransporter-1, driving neuronal Cl⁻ accumulation and subsequent osmotic edema. The swelling process further involves ammonia-induced upregulation of Aquaporin-4 coupled with Kir4.1 and connexin 43 inhibition, collectively disrupting hydroionic and osmotic homeostasis (Braissant et al. 2013). Concurrent reduction in glial fibrillary acidic protein expression compromises structural resistance to osmotic stress, exacerbating astrocytic edema (Felipo and Butterworth 2002).

Collectively, these mechanisms underscore the critical role of mitochondrial dysfunction, inflammation and neurotransmitter system dysregulation in ammonia-induced neurotoxicity.

#### Lactate

##### Disruption of the astrocyte-neuron lactate shuttle

The brain exhibits enormous energy demands predominantly driven by neuronal activity, prompting the evolution of specialized intercellular metabolic coupling (Wu et al. 2023). Astrocytes primarily metabolize glucose via glycolysis, while neurons preferentially utilize lactate for oxidative ATP generation (Prasad et al. 2024). Brain lactate originates dually: a minor fraction enters from peripheral circulation by crossing the BBB through monocarboxylate transporter 1, while the majority is produced locally through the astrocyte-neuron lactate shuttle (Wang et al. 2024). Within this shuttle system, astrocytes convert glucose to lactate through glycolysis and release it into the extracellular space, where it is taken up by neurons (Wu et al. 2023). Neurons then oxidize lactate through the TCA cycle to support activity-dependent ATP demands. Notably, MMA is a potent inhibitor of LDH, particularly blocking the conversion of lactate to pyruvate (Saad et al. 2006). This inhibition disrupts the astrocyte-neuron lactate shuttle, compromising neuronal energy metabolism.

##### Biomarker utility and neurological dysfunction

Pathological hyperlactatemia is clinically associated with increased mortality risk in critically ill adults and neonates (Achanti and Szerlip 2023; Matsushita et al. 2023). Urinary lactate serves as both an early diagnostic biomarker for hypoxic-ischemic encephalopathy in term infants and a strong predictor of long-term neurodevelopmental outcomes in preterm brain injury, potentially mediated by impaired cerebral energy metabolism during early neurodevelopment (Zasada et al. 2025). Magnetic resonance spectroscopy further indicates that lactate accumulation in deep gray matter structures, particularly when measured as the lactate/N-acetylaspartate ratio, predicts adverse neurodevelopmental outcomes after neonatal encephalopathy (Thayyil et al. 2010). Experimental studies demonstrate that lactate induces seizures and cognitive deficits in animal models through impaired synaptic transmission, disrupted neuronal network oscillations, and mitochondrial dysfunction (Kann 2024; Xie et al. 2024). Collectively, these findings establish lactate as a key biomarker and pathological mediator of adverse neurological outcomes.

##### Neurotoxic and neuroprotective role

Lactate functions as an energy substrate, particularly under conditions of low metabolic demand or glucose insufficiency. However, elevated lactate levels may competitively inhibit glucose transport, impairing neuronal energy supply. Additionally, lactate attenuates presynaptic release of glutamate and GABA, suggesting that an increased lactate/glucose ratio promotes central fatigue via energy depletion and neurotransmitter dysregulation (Kann 2024). Lactate accumulation also elevates ROS, inducing lipid peroxidation, protein oxidation, and DNA damage (Wang et al. 2024; Jia et al. 2021). Furthermore, it depletes GSH, exacerbating oxidative stress by compromising antioxidant defenses (Lewerenz et al. 2010). In neurodegenerative diseases, microglia-derived lactate enhances pro-inflammatory cytokine release (e.g., TNF-α, IL-6 and IL-1β) (Yang et al. 2024a). Paradoxically, exogenous lactate suppresses LPS-induced inflammation via NLRP3 inflammasome inhibition (Liang et al. 2024). Notably, a study demonstrated that lactate—not ammonia-derived glutamine—acts as the key osmotic driver of cerebral edema in rodent models (Bosoi et al. 2014). Collectively, these findings indicate that central lactate accumulation contributes to neural dysfunction and injury. However, targeting lactate reduction as a therapeutic strategy may be unwise, given its vital physiological roles in energy production, neurodevelopment, signal transduction, and maintenance of brain homeostasis. Owing to its high energy demand and limited substrate reserves, the brain relies heavily on lactate as an indispensable energy source that cannot be fully replaced by glucose (Li et al. 2022). Experimental evidence underscores lactate's therapeutic potential. In traumatic brain injury model, inhibition of endogenous lactate synthesis exacerbates cerebral energy deficits and neuronal damage, whereas exogenous lactate supplementation attenuates cognitive impairment (Chen et al. 2000; Holloway et al. 2007). Another study highlighted the critical role of lactate in long-term memory formation (Suzuki et al. 2011). Mechanistically, lactate activates SIRT1, upregulates brain-derived neurotrophic factor expression, and enhances cognition, learning, and memory (El Hayek et al. 2019). Moreover, lactate exerts neuroprotective effects through G protein-coupled receptor GPR81 activation, including promoting cerebral energy supply, attenuating neuroinflammation and enhancing neurovascular coupling (Li et al. 2023). Beyond its canonical roles as an energy substrate and gluconeogenic precursor, lactate regulates mitochondrial function, modulates neuroinflammation, and interacts with neurotransmitter systems (Yang et al. 2024a; Li et al. 2023).

### Post-translational modification (PTM)

Accumulating evidence establishes the pivotal role of metabolite-driven toxicity in MMAemia-associated neuropathology. However, circulating toxins alone cannot fully account for the observed neurological damage, implicating additional pathogenic mechanisms (Head et al. 2023). PTMs represent a fundamental regulatory layer controlling protein function through dynamic alterations of activity, stability, and interaction networks (Lee et al. 2023). Crucially, these modifications possess the capacity to transform entire metabolic pathways or cellular processes, thereby playing an instrumental role in disease pathogenesis (Lossi et al. 2024). In MMAemia, MMUT deficiency generates a distinct biochemical milieu with mitochondrial acyl-CoA imbalances, driving aberrant non-enzymatic lysine hyperacylation (particularly propionylation and methylmalonylation) (Head et al. 2023, 2022). Proteomic studies revealed widespread hyperacylation in key metabolic pathways in both MMA patients and murine models, encompassing: (1) central carbon metabolism (TCA cycle, glycolysis/gluconeogenesis); (2) nitrogen metabolism (urea cycle, amino acid metabolism); (3) lipid metabolism (β-oxidation, fatty acid metabolism); and (4) redox homeostasis systems (glutathione metabolism, oxidative stress response) (Head et al. 2022). Furthermore, PA induces epigenetic dysregulation via histone acetylation in neurons/astrocytes, potentially modifying gene expression profiles linked to neurotoxicity (Morland et al. 2018; Nguyen et al. 2007). Lactylation, a novel PTM involving the covalent conjugation of lactate moieties to histone lysine residues, exists in both enzymatically catalyzed and non-enzymatic forms (Li et al. 2023). This epigenetic mechanism has been conclusively shown to directly modulate chromatin architecture and transcriptional activity (Wang et al. 2024; Sharma and Pal 2021). Accumulating evidence increasingly highlights the vital role of lactylation in modulating the activity of glycolytic enzymes, the level of neuronal excitability, the intensity of inflammatory responses, and the progression of neurodevelopmental processes (Hagihara et al. 2021; Chen et al. 2025). Consequently, MMAemia-related neuropathology manifests through dual mechanisms: direct metabolite toxicity (primary and secondary metabolites) and aberrant PTMs (notably methylmalonylation, propionylation, and acetylation). These modifications collectively reprogram cellular metabolism and redox states, synergizing with neuroinflammation and excitotoxicity to drive progressive neurological damage.

Although multiple PTMs have been identified in MMAemia, their precise pathophysiological contributions and therapeutic potential remain poorly understood. The concurrent existence of metabolite-driven neurotoxicity and PTMs-mediated dysregulation suggests that mechanistic elucidation of PTMs-related neurotoxicity could identify novel targetable pathways and advance therapeutic development for this disorder.

### Convergent mechanisms of cerebral injury in MMAemia

In MMAemia, the cumulative effects of these metabolites converge on: mitochondrial dysfunction, neuroinflammation and excitotoxicity (Table 1).

**Table 1:** Mechanisms of metabolite-induced brain injury in methylmalonic acidemia

Mechanism	Metabolite	Molecular Mechanisms and Signaling Pathway	References
Energy depletion	MMA	Inhibits TCA cycle enzymes (SDH/KGDHC), MRC (I/II/II-III) and metabolic enzymes (LDH/pyruvate carboxylase/β-hydroxybutyrate dehydrogenase)	(Kolker and Okun 2005; Brusque et al. 2002; Jafari et al. 2013)
		Disrupts malate-aspartate shuttle	(Kolker and Okun 2005)
		Induces coenzyme Q10 deficiency	(Proctor et al. 2020)
	2-MCA	Inhibits TCA cycle (citrate synthase/aconitase/isocitrate dehydrogenase); suppresses GDH	(Kölker et al. 2013; Amaral et al. 2016)
	PA	Inhibits metabolic enzymes (PDH/KGDHC/succinyl-CoA ligase/NAGS/Na^+^,K^+^-ATPase) and MRC III	(Kölker et al. 2013; Morath et al. 2008; Wyse et al. 1998)
	Hcy	Inhibits MRC I/II/IV/V and Na^+^,K^+^-ATPase	(Kolling et al. 2016; Cordaro et al. 2021)
	Ammonia	Inhibits TCA cycle enzymes (KGDH/isocitrate dehydrogenase/malate dehydrogenase), MRC II/IV and PDH	(Ribas et al. 2022; Prasad et al. 2024; Heidari 2019; Sorensen et al. 2024)
	Lactate	Inhibits glucose transport	(Kann 2024)
Oxidative stress	MMA	↑ROS/RNS	(Tejero et al. 2024; Schuck et al. 2013; Melo et al. 2011; Colín-González et al. 2015; Klemmensen et al. 2024)
		Induces mitochondrial permeability transition	(Melo et al. 2011; Klemmensen et al. 2024)
		Induces oxidative damage to protein/lipid/DNA	(Fernandes et al. 2011; Pettenuzzo et al. 2003; Schuck et al. 2013; Colín-González et al. 2015; Fontella et al. 2000; Andrade et al. 2014)
		Depletes antioxidant defenses (↓GPx/GSH/SOD)	(Fernandes et al. 2011; Pettenuzzo et al. 2003; Schuck et al. 2013; Fontella et al. 2000; Viegas et al. 2014; Almeida et al. 2022)
	2-MCA	Induces mitochondrial permeability transition pore opening; depolarizes membrane potential; triggers matrix swelling	(Amaral et al. 2016)
	PA	↑ROS → lipid peroxidation and protein carbonylation	(Fontella et al. 2000; Rigo et al. 2006)
		Disrupts MAPK/ERK-mediated autophagic flux	(Choi et al. 2020)
		Depletes antioxidant defenses (↓GPx/GSH/SOD/CAT)	(Fernandes et al. 2011; El-Ansary et al. 2012; Khalil et al. 2015)
		Suppresses Nrf2/HO-1 pathway	(Erten 2021)
	Hcy	Depletes antioxidant defenses (↓GPx/SOD/CAT)	(Longoni et al. 2018; Kolling et al. 2016; Cordaro et al. 2021)
	Ammonia	↑ROS/RNS/NO → protein tyrosine nitration/lipid peroxidation/S-nitrosylation of cysteine residues/nucleic acid oxidation	(Redolfi-Bristol et al. 2024; Angelova et al. 2022; Skowronska and Albrecht 2013)
		Depletes antioxidant defenses (↓GPx/GSH/SOD/CAT)	(Heidari 2019)
	Lactate	↑ROS → oxidative damage (lipids/proteins/nucleic acids)	(Wang et al. 2024; Jia et al. 2021)
		Depletes GSH	(Lewerenz et al. 2010)
Neuro-inflammation	MMA	Activates microglia/astrocytes	(Gabbi et al. 2017; Almeida et al. 2022; Li et al. 2017)
		↑IL-1β/IL-6/TNF-α	(Li et al. 2021, 2017; Gabbi et al. 2017; Ribeiro et al. 2013; Dos Reis et al. 2024)
		Activates SUCNR1 → MAPK/ERK/PKC signaling → NF-κB/p38 activation	(Tejero et al. 2024; Geubelle et al. 2017; Gilissen et al. 2016)
	PA	Activates NF-κB; ↑TNF-α/IL-1α/6/8	(El-Ansary et al. 2012; Erten 2021)
	Hcy	Activates microglia/astrocytes	(Longoni et al. 2018; Yin et al. 2025; Yang et al. 2024b)
		Activates NF-κB and suppresses HO-1 → ↑cytokines (TNF-α, IL-1β and IL-6), chemokines, and prostaglandin E2	(Djuric et al. 2018; Longoni et al. 2018)
		Activates NLRP3/caspase-1/GSDMD axis	(Yin et al. 2025; Yang et al. 2024b)
	Ammonia	Activates microglia/astrocytes	(Claeys et al. 2025; Jo et al. 2021)
		↑IL-1β/6/TNF-α/iNOS/high-mobility group box 1/cyclooxygenase-2	(Gabbi et al. 2018; Bobermin et al. 2020)
		Activates p38 MAPK and NF-κB pathways	(Bobermin et al. 2020)
	Lactate	↑TNF-α/IL-1β/6	(Yang et al. 2024a)
Excitotoxicity	MMA	Disrupts glutamate metabolism; overstimulates NMDA receptors; inhibits GAD → ↓GABA; alters AChE activity	(Brusque et al. 2001; De Mattos-Dutra et al. 2000; Affonso et al. 2013)
	PA	Activates NMDA receptor; inhibits GABA transaminase → ↑extracellular GABA	(Rigo et al. 2006; Morland et al. 2018)
	Hcy	Activates NMDA and group I metabotropic glutamate receptors	(Luzzi et al. 2022; Djuric et al. 2018; Zieminska et al. 2003)
	Ammonia	Disrupts glutamate/GABA-glutamine cycle (↑glutamine/↓GABA)	(Royes et al. 2016)
		Dysregulates NMDA/AMPA/mGlu receptors	(Oja et al. 2017; Braissant et al. 2013)
		Induces synaptic dysfunction	(Oja et al. 2017)
		Dysregulates neurotransmitter systems (serotonergic/cholinergic/dopaminergic/noradrenergic pathways)	(Lee and Kim 2022; Oja et al. 2017)
	Lactate	Inhibits presynaptic release of glutamate and GABA	(Kann 2024)
PTM	MMA, PA	Methylmalonylation and propionylation disrupt key metabolic pathways: central carbon (TCA cycle, glycolysis), nitrogen (urea cycle, amino acids), lipid (β-oxidation, fatty acids), and redox (glutathione, oxidative stress)	(Head et al. 2022)
	PA	Induces histone acetylation → modifies gene expression profiles	(Morland et al. 2018; Nguyen et al. 2007)
	Lactate	Lactylation modulates the glycolytic, neuronal excitability, neuroinflammation, and neurodevelopment	(Hagihara et al. 2021; Chen et al. 2025)
Neuronal injury	MMA	Activates MAPK and p53 pathway	(Zhou et al. 2024; Han et al. 2015)
		↓ATP/ADP → ↑ROS → mitochondrial membrane depolarization → ↓Bcl-2, ↑Bax	(Zhou et al. 2024; Mclaughlin et al. 1998; Li et al. 2014)
		Activates caspases-1/3/8	(Gabbi et al. 2017)
	PA	Reduces dendritic spine density → synaptic dysfunction	(Lobzhanidze et al. 2020)
		↓Bcl-2, ↑caspase-3	(Khalil et al. 2015)
	Hcy	Activates ERK/p38 MAPK → ↑caspase-3/9; activates p53 pathway → ↑Bax → ↑caspase-3	(Ivanova et al. 2020)
Neuroglial dysfunction	MMA	Activates microglia/astrocytes	(Gabbi et al. 2017; Almeida et al. 2022; Li et al. 2017)
	2-MCA	Causes astrocytic swelling; disrupts oligodendrocyte maturation; induces glial apoptosis	(Jafari et al. 2013)
	PA	Rearranges astrocytic cytoskeleton (actin/glial fibrillary acidic protein)	(De Almeida et al. 2006)
	Hcy	Induces astrocyte reactivity and microglial ferroptosis/pyroptosis	(Longoni et al. 2018; Yin et al. 2025; Yang et al. 2024b)
	Ammonia	Activates Na^+^-K^+^−2Cl^−^ cotransporter-1 → Cl⁻ accumulation; ↑AQP4/Kir4.1, ↓connexin 43 → hydroionic and osmotic homeostasis disruption; reduces glial fibrillary acidic protein → compromises structural integrity → astrocytic edema	(Felipo and Butterworth 2002; Braissant et al. 2013; Eid and Lee 2013)
Neurovascular matrix disruption	Hcy	Impairs NO-mediated endothelial vasodilation	(Lai and Kan 2015)
		Induces endothelial death (apoptosis/pyroptosis/ferroptosis), vascular smooth muscle cell proliferation and ECM remodeling → ↑vascular permeability	(Lai and Kan 2015; Ortiz-Salguero et al. 2024)
		Activates platelet and inhibits antithrombin III → thrombosis	(Hainsworth et al. 2016)
		Activates MMP-9, degrades tight junction proteins(claudin-5/occludin)	(Zhong et al. 2019; Tawfik et al. 2021)
	Ammonia	Disrupts cerebral blood flow autoregulation	(Bjerring et al. 2009)

*Abbreviations*: *AChE* acetylcholinesterase, *ERK* extracellular signal-regulated kinase, *GSDMD* gasdermin D, *HO-1* heme oxygenase-1, *MAPK* mitogen-activated protein kinase, *MMP* matrix metalloproteinase, *NLRP3* NLR family pyrin domain containing 3, *Nrf2* nuclear factor erythroid 2-related factor 2, *PKC* protein kinase C, *SUCNR1* succinate receptor 1

#### Mitochondrial dysfunction: metabolic disruption, energy crisis and oxidative stress

MMAemia is caused by defects in MMUT or its coenzyme Cbl, leading to toxic accumulation of primary metabolites (MMA, 2-MCA, PA, and Hcy) and secondary metabolites (ammonia and lactate) (Longo et al. 2022; Haijes et al. 2019). These metabolites inhibit the urea cycle and aerobic oxidation processes. As a result, blood ammonia and lactate levels increase (Ling et al. 2025). Moreover, they inhibit multiple enzymes involved in the TCA cycle, OXPHOS, and MRC complexes (Fig. 1). By obstructing mitochondrial respiration, they impede the production of ATP, thus disrupting the energy supply to cells. Notably, the energy demand of brain activity is dynamically variable. Astrocytes, for example, produce L-lactate through aerobic glycolysis to provide energy for working neurons, a process termed the astrocyte-neuron lactate shuttle that highlights metabolic coupling between glia and neurons during synaptic activity (Virtuoso et al. 2021). In the case of energy shortage, astrocytes in different brain regions utilize different substances for energy production. Specifically, astrocytes in the striatum use fatty acids, and those in the cerebellum use amino acids, but this may lead to oxidative stress (Hu and Kong 2022; Virtuoso et al. 2021). Mitochondria are organelles that are primarily known for ATP production through OXPHOS. They also play crucial roles in integrating energy metabolism, maintaining redox balance, modulating apoptosis and mitophagy, regulating inflammatory signaling, and influencing cell fate decisions. The human brain exhibits diverse mitochondrial phenotypes, including variations in OXPHOS enzyme activities, mitochondrial DNA content, volume density, and respiratory capacity, which are shaped by distinct cell types. Consequently, high-energy-demanding cells exhibit greater reliance on mitochondrial network stability, rendering them especially vulnerable to its dysregulation (Mosharov et al. 2025). In MMAemia patients and animal models, mitochondrial structural abnormalities (including mitochondrial swelling and disorganized cristae) are observed across multiple tissues including the liver, kidney, heart, and skeletal muscle (Kölker et al. 2013; Zsengeller et al. 2014; Wilnai et al. 2014). Moreover, lysosomal-autophagy and the PINK1/Parkin-mediated mitophagy pathway are significantly impaired in MMAemia, compromising the recognition and elimination of damaged mitochondria (Costanzo et al. 2024; Luciani et al. 2020; Luciani and Devuyst 2020). Consequently, pathological accumulation of aberrant mitochondria exacerbates mitochondrial dysfunction. The accumulated metabolites inhibit MRC complexes, leading to electron transport disruption, impaired proton gradient generation, and a consequent reduction in the mitochondrial membrane potential. These defects promote electron leakage and subsequent ROS generation, initiating oxidative stress (Luciani et al. 2021; Wajner and Goodman 2011). Notably, the metabolites also concurrently deplete both enzymatic (e.g., SOD and CAT) and non-enzymatic antioxidant defenses, creating a redox imbalance that induces oxidative damage to cellular macromolecules including proteins, lipids, and nucleic acids, ultimately compromising normal cellular function (El-Ansary et al. 2012; Longoni et al. 2018). Furthermore, oxidative stress activates multiple stress-responsive signaling pathways and alters gene expression profiles, which further disrupts cellular metabolism and physiological processes. This self-perpetuating cycle of mitochondrial damage and oxidative injury progressively worsens mitochondrial pathology, constituting a key mechanistic driver of MMA-associated neurological damage (Dimitrov et al. 2021).

#### Neuroinflammation: glial activation and cytokine release

Neuroinflammation, a hallmark of CNS pathology, is characterized by microglial activation, aberrant cytokine release, immune cell infiltration, and BBB disruption (Ribeiro et al. 2013). In MMAemia, mitochondrial dysfunction disrupts the TCA cycle and OXPHOS, leading to excessive ROS production. These ROS serve as signaling molecules that activate NF-κB and other inflammatory pathways, thereby inducing the release of IL-1β, IL-6, and TNF-α. These cytokines not only recruit peripheral immune cells but also potentiate NF-κB activation, forming a vicious cycle (Denley et al. 2025; Cheataini et al. 2023; Shabab et al. 2017). Concurrently, mitochondrial membrane damage causes leakage of mitochondrial proteins, which are recognized as danger-associated molecular patterns, exacerbating neuroinflammation (Cheataini et al. 2023). The resulting oxidative and nitrosative stress (ROS/RNS) further compromises BBB integrity, allowing endogenous toxins and inflammatory mediators to infiltrate the CNS. This activates microglia, which release additional neurotoxic cytokines and oxidative molecules, depleting antioxidant defenses (Li et al. 2021; Tejero et al. 2024). Under inflammatory conditions, astrocytes undergo alterations in mitochondrial dynamic and activate autophagy to maintain mitochondrial function. Notably, activated microglia exhibit a metabolic shift toward glycolysis (a Warburg-like effect), which amplifies their pro-inflammatory phenotype while impairing mitochondrial function through bidirectional crosstalk between metabolism and inflammation (Zhang et al. 2021; Harry et al. 2020).

#### Excitotoxicity: Neurotransmitter imbalance and synaptic dysfunction

Excitotoxicity in MMAemia arises from glutamate/GABA‒glutamine metabolic reprogramming, which alters neuronal excitability and involves altered glutamatergic synapses, ion channel activity, and synaptic transmission (Denley et al. 2025). The accumulation of neurotoxic metabolites perturbs neurotransmitter systems, particularly NMDA, GABA, and AMPA receptors, leading to elevated synaptic glutamate and pathological NMDA receptor overactivation, which subsequently: (1) disrupts neuronal signaling and neurotransmitter balance (Brusque et al. 2001; Rigo et al. 2006; Morland et al. 2018; Braissant et al. 2013); (2) induces excessive Ca^2+^ influx, leading to mitochondrial Ca^2+^ overload, ATP depletion, and apoptotic activation via cytochrome c release (Ji et al. 2019; Oja et al. 2017); and (3) triggers pro-inflammatory cytokine release, which promotes neuroinflammation and BBB dysfunction (Ji et al. 2019). This excitotoxic cascade is further exacerbated by oxidative stress-mediated attenuation of action potentials and impaired ion channel activity, while ATP depletion causes membrane potential collapse and pathological hyperexcitability (Denley et al. 2025). Notably, cytokines such as TNF-α and IL-1β amplify NMDA receptor-mediated Ca^2^⁺ influx, with TNF-α additionally inhibiting astrocytic glutamate uptake, thereby exacerbating neuronal excitotoxicity and synaptic dysfunction in MMAemia neuropathology (Gabbi et al. 2017).

**Fig. 2:**
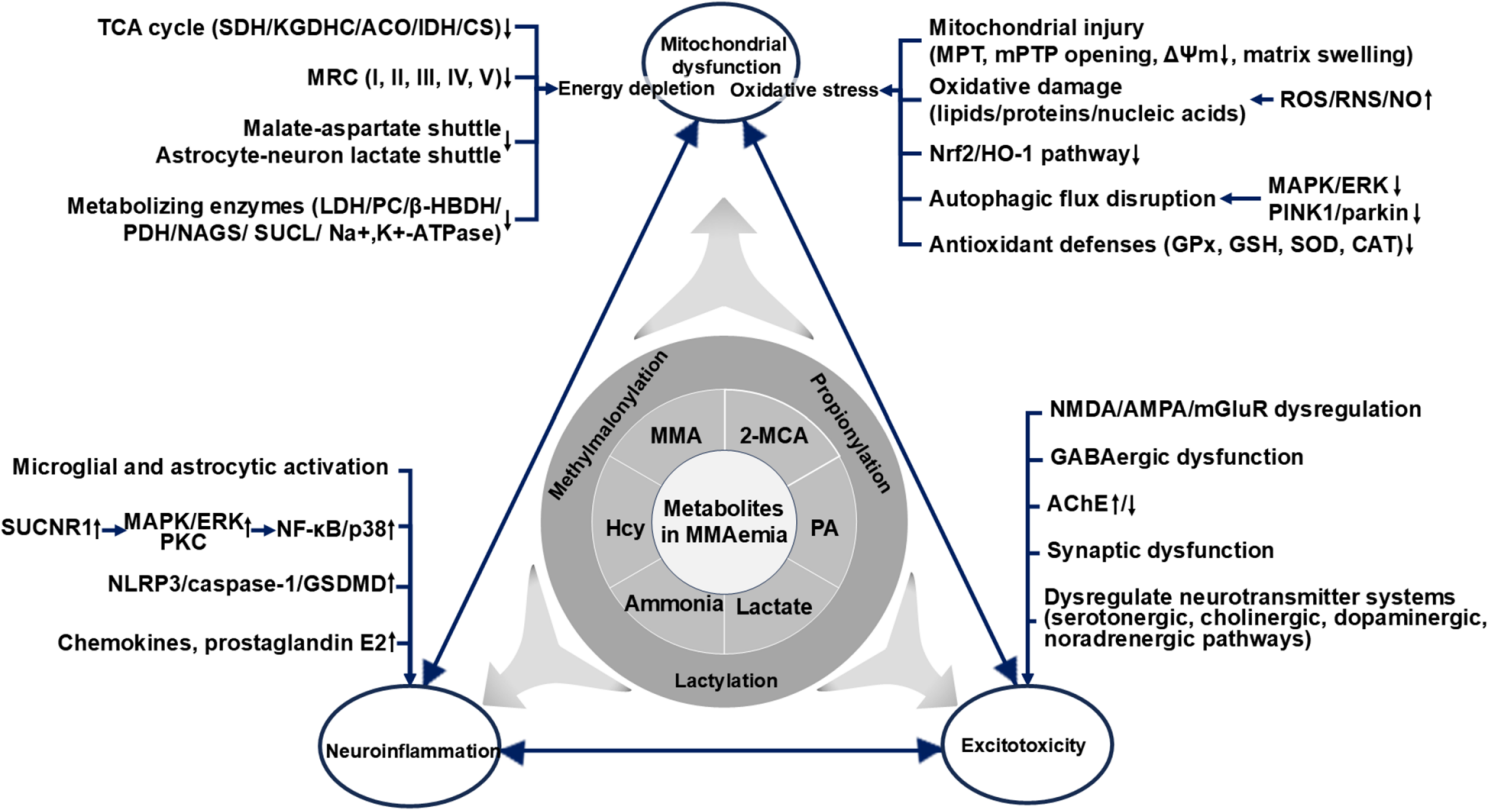
Pathogenic mechanisms of brain injury in MMAemia mediated by toxic metabolites. This diagram illustrates how metabolites (MMA, 2-MCA, PA, Hcy, ammonia and lactate) and their driven PTMs (methylmalonylation, propionylation and acetylation) concertedly act on three core pathological processes—mitochondrial dysfunction, neuroinflammation, and excitotoxicity—to collectively induce brain injury in MMAemia. Abbreviations: ACO, aconitase; AChE, acetylcholinesterase; β-HBDH, β-hydroxybutyrate dehydrogenase; CS, citrate synthase; ERK, extracellular signal-regulated kinase; HO-1, heme oxygenase 1; MAPK, mitogen-activated protein kinase; MPT, mitochondrial permeability transition; mPTP, mitochondrial permeability transition pore; NF-κB, nuclear factor κB; Nrf2, nuclear factor erythroid 2-related factor 2; PC, pyruvate carboxylase; PKC, protein kinase C; SUCL, succinyl-CoA ligase; SUCNR1, succinate receptor 1; ΔΨm, mitochondrial membrane potential

The spatial and temporal coordination of cellular and molecular components is a fundamental feature of biological systems, and the CNS exemplifies this principle. Isolated knowledge of individual elements (e.g., neurons, glia, or vascular cells) is insufficient to predict long-term physiological adaptations or pathological cascades (Virtuoso et al. 2021). Neuronal activity and synaptic plasticity depend on coordinated interactions among neural cells and extracellular components. Astrocytes regulate neurovascular coupling, BBB integrity, and synaptic homeostasis, while microglia mediate synaptic pruning and immune surveillance. Oligodendrocytes support myelination and synaptic plasticity, with ECM proteins such as thrombospondins facilitating synaptogenesis. The neurovascular unit ensures metabolic support and waste clearance, where pericytes control BBB permeability. Together, these interconnected mechanisms, metabolic-energetic regulation (energy demands, redox balance), neuroimmune crosstalk (pro-inflammatory/anti-inflammatory balance), neural excitation-inhibition equilibrium, and PTMs (e.g., methylmalonylation, propionylation and lactylation), in conjunction with glial networks, neurovascular coupling, and ECM remodeling collectively maintain CNS homeostasis. In MMAemia, toxic metabolites (e.g., MMA, 2-MCA, PA, Hcy, ammonia, lactate) serve as primary triggers of cerebral injury. These metabolites preferentially target mitochondria, leading to dysfunction characterized by energy depletion—due to impaired OXPHOS and TCA cycle activity—and oxidative stress resulting from excessive ROS production and antioxidant depletion. The ensuing mitochondrial damage initiates neuroinflammation through ROS/NF-κB and succinate receptor-mediated pathways, and excitotoxicity via glutamate accumulation and NMDA receptor overactivation. These processes act in a feed-forward manner, amplifying neuronal injury through reciprocal reinforcement (Fig. 2).

### Targeting metabolic dysfunction and neurotoxicity in MMAemia

**Fig. 3:**
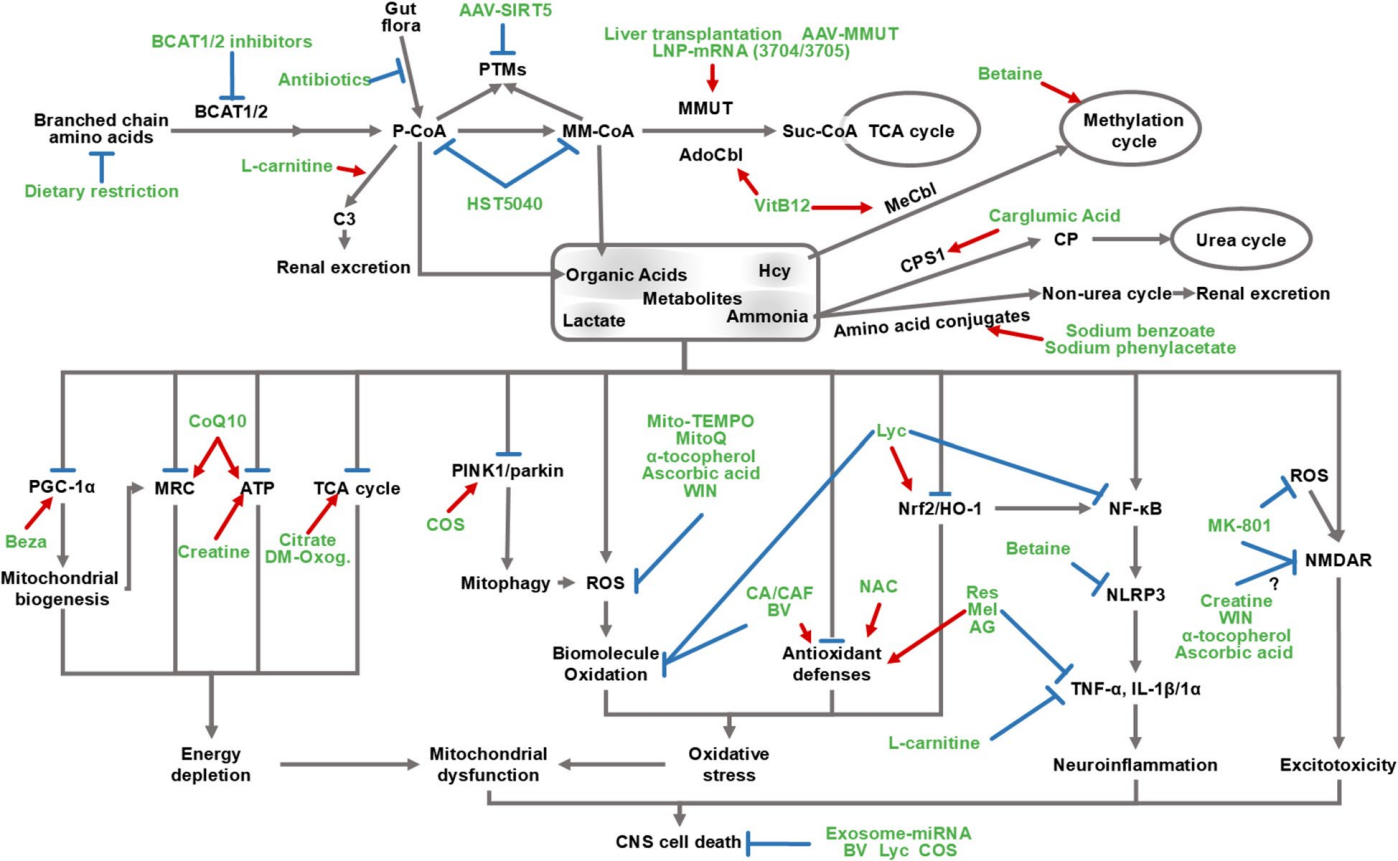
Therapeutic strategies targeting metabolic and cellular dysfunctions in MMAemia-induced brain injury. This schematic presents an integrated therapeutic framework targeting both metabolic and cellular dysfunction pathways in MMAemia-associated neuropathology. Red arrows indicate activation, and blunt blue arrows represent inhibition of key pathological processes. Therapeutic interventions are highlighted in green text. The therapeutic strategies specifically address five core pathological domains: metabolic pathway regulation, mitochondrial function (targeting both energy depletion and oxidative stress), neuroinflammation suppression, excitotoxicity mitigation, and CNS cell death prevention. Collectively, this framework highlights the multimodal therapeutic approach required to restore metabolic homeostasis and cellular function in MMAemia-associated neurological pathologies. Abbreviations: AG, aminoguanidine; BCAT1/2, branched-chain amino acid transaminase 1/2; Beza, bezafibrate; BV, bee venom (apitoxin); CA, caffeic acid; CAF, caffeine; CoQ10, coenzyme Q10; COS, costunolide; CP, carbamoyl phosphate; C3, propionyl-L-carnitine; DM-Oxog., dimethyl-oxoglutarate; HO-1, heme oxygenase 1; HST5040, 2,2-dimethylbutanoic acid; LNP-mRNA, lipid nanoparticle-encapsulated messenger RNA; Lyc, lycopene; Mel, melatonin; MK-801, dizocilpine maleate; MM-CoA, methylmalonyl-CoA; NAC, N-acetylcysteine; PGC-1α, peroxisome proliferator-activated receptor gamma coactivator 1-α; PINK1, PTEN-induced kinase 1; Res, resveratrol; Suc-CoA, succinyl-CoA; WIN, WIN 55,212–2 (cannabinoid receptor agonist)

This section examines mechanism-based therapeutic strategies, encompassing conventional metabolic management, emerging gene therapies, and neurotoxicity-targeted interventions, to address systemic metabolic dysregulation and mitigate associated CNS injury in MMA (Table 2 and Fig. 3).

**Table 2:** Potential therapeutic strategies for brain injury in Methylmalonic Acidemia

Target Category	Therapy	Subject Type	Study Design	Mechanism of Action	Limitations
Propionate Oxidation Pathway	Dietary restriction	Humans	Recommended by clinical guidelines	Restricts branched-chain amino acid and odd-chain fatty acid intake; reduces propionyl-CoA and methylmalonyl-CoA accumulation	Ineffective against endogenous/gut-derived metabolites
	BCAT1/2 inhibitors	Patient-derived MMA hepatocytes (from liver transplant recipients) and Mmut p.L690Ins/p.L690Ins mice	A combined in vitro and in vivo study	Blocks branched-chain amino acid catabolism	Limited effects on phenotype, core pathology and reducing marker concentrations
	Short-term antibiotics (metronidazole/neomycin)	Humans	Recommended by clinical guidelines	Reduces gut microbiota-derived propionate production	Only reduces gut-derived propionate; no effect on endogenous metabolites
	L-carnitine	Humans	Prospective observational study	Restores carnitine pool; enhances propionylcarnitine excretion; lowers TNF-α/CRP; normalizes neurodegenerative biomarkers (NCAM-1/cathepsin-D); attenuates oxidative stress (↓lipid/protein oxidation)	Unclear long-term efficacy and safety
	Cobalamin	Humans	Recommended by clinical guidelines	Activates residual MMUT to convert methylmalonyl-CoA to succinyl-CoA	Only effective in B12-responsive patients; does not fully normalize metabolite levels
	Betaine	Sprague–Dawley rats, HMC3 cells and SH-SY5Y cells	A combined in vitro and in vivo study	Promotes BHMT expression, enhances methylation, and reduces Hcy levels. Inhibits microglial pyroptosis and the NLRP3/caspase-1/GSDMD pathway	Only targets Hcy metabolism; does not fully normalize metabolite levels
	Liver transplantation	Humans	Systematic review and meta-analysis	Restores hepatic MMUT activity	High surgical risk; uncertain neuroprotective efficacy; variable long-term outcomes
	HST5040(2,2-dimethylbutyrate)	Primary hepatocytes derived from patients, CD-1 mouse and neonatal Göttingen minipigs	A combined in vitro and in vivo study	Redistributes CoA pool, reduces propionyl-CoA/methylmalonyl-CoA; lowers ammonia; improves mitochondrial function	Phase II trial (NCT04732429; terminated Jan 2024-undisclosed reasons)
Ammonia Metabolism	Sodium benzoate/phenylacetate	Humans	Recommended by clinical guidelines	Conjugates with glycine/glutamine to enhance urinary ammonia excretion	No significant reduction in organic acid levels
	Carglumic Acid	Humans	Clinical observational studies, randomized controlled trials, and expert experience summaries	Acts as an NAG analog to allosterically activate CPS1, thereby enhancing urea cycle activity and promoting ammonia detoxification; improves neurological symptoms (disorders of consciousness)	Direct neuroprotective effects against long-term CNS damage remain to be fully elucidated
Gene Therapy	AAV-mediated *MMUT* gene delivery	Mice	In vivo experimental study	In *Mut*^−/−^ mice, AAV8-*MMUT* restores hepatic MMUT activity, reduces systemic MMA concentrations, and mitigates neuropathological damage; AAV9 crosses the BBB, decreasing cerebral MMA accumulation	Preclinical (animal models); immunogenicity risks; limited CNS bioavailability (BBB impermeability); unestablished long-term safety profile
	AAV-mediated *SIRT5* gene delivery	Mice	In vivo experimental study	Reactivates *SIRT5*-mediated removal of malonyl/methylmalonyl groups from lysine residues	No direct reduction in systemic MMA levels; potential functional redundancy from other sirtuins
	LNP-encapsulated mRNA-3704/3705	Human fibroblasts, HeLa cells, Sprague Dawley rats, and 2 Mut^−^/^−^ MMA mouse models	A combined in vitro and in vivo study	Encodes functional MMUT enzyme, restoring methylmalonyl-CoA metabolism	Phase I/II trials (NCT03810690, NCT04899310, NCT05295433); optimal dosing undetermined; limited brain delivery efficiency; long-term immunogenicity unclear
	Gene editing	Mice	In vivo experimental study	Repair *MMUT* mutations	Immunogenicity; unconfirmed long-term safety; low delivery efficiency; biallelic requirement
Energy Restoration	Disodium citrate, dimethyl-oxoglutarate	MMUT-knockout 293 T cells, primary hepatocytes, and primary fibroblasts derived from MMA patients	In Vitro Laboratory Study	Restores TCA flux	Partial metabolic rescue demonstrated in vitro; lacks in vivo validation
	Creatine	Wistar rats	In vivo experimental study	Enhances cerebral energy via phosphocreatine/ATP pathway; prevents lactic acidosis; attenuates NMDA-mediated excitotoxicity	Under preclinical evaluation (animal data)
	CoQ10	Mut^−^/^−^ mice	In vivo experimental study	Restores MRC II-III activity and preserves ATP synthesis	In vitro only
	Bezafibrate	C6 rat glioma cells	In Vitro Laboratory Study	PPARγ agonist that upregulates PGC-1α-dependent mitochondrial biogenesis, enhancing respiratory chain efficiency	In vitro only
Antioxidant	Costunolide	Rats and PC12 cells	A combined in vitro and in vivo study	Activates PINK1/Parkin-mediated mitophagy; ↓ROS, ↓MDA, ↑SOD; attenuates neuronal apoptosis and cognitive impairment	Rodent efficacy demonstrated; human safety/BBB delivery unconfirmed
	Mito-TEMPO/MitoQ	Primary renal tubular epithelial cells and mmut-deficient zebrafish	A combined in vitro and in vivo study	Scavenges mitochondrial ROS	Evaluated only in ex vivo patient renal cells and *MMUT*-knockout zebrafish models; no brain injury therapeutic assessment
	N-acetylcysteine	NA	NA	Enhances glutathione biosynthesis and improves oxidative stress	Not tested in MMAemia models
	Ascorbic acid	Wistar rats	In vivo experimental study	Exerts ROS-scavenging activity; suppresses lipid peroxidation and ROS-induced excitotoxicity; mitigates seizure severity and cognitive impairments	Preclinical (animal models); limited BBB penetration
	α-tocopherol	Rat brain synaptosomes; Wistar rats	In vivo and vitro experimental study	Inhibits membrane lipid peroxidation; reduces free radical-mediated excitotoxicity; decreased seizure duration	Preclinical (animal models); limited BBB penetration and potential interactions with other lipid-soluble compounds
	WIN 55,212–2	Rat brain synaptosomes	In vitro experimental study	Cannabinoid receptor agonism to scavenge ROS and inhibit lipid peroxidation; NMDA receptor modulation to reduce excitotoxicity	Preclinical (animal models); Potential psychoactive effects; Poor BBB penetration
	Caffeic acid/caffeine	Drosophila melanogaster	In vivo experimental study	Inhibits lipid peroxidation, ↑SOD/CAT, replenishes energy substrates; improves neurobehavioral deficits	Preclinical (animal models); limited BBB penetration; potential AChE modulation
	Apitoxin	Sprague–Dawley rats	In vivo experimental study	Inhibits lipid/protein/DNA oxidation; ↑SOD/GSH/CAT; antiapoptotic (↑Bcl-2, ↓Caspase-3)	Preclinical (animal models); immunogenicity risk; uncertain dosing; unproven long-term safety/efficacy
Anti-inflammatory/antioxidant	Melatonin	C6 astroglial cells; rat brain synaptosomes	In Vitro Laboratory Study	Inhibits TNF-α, IL-1β; restores GSH homeostasis; reduces lipid peroxidation	Limited to in vitro data; circadian disruption risk; neurorestorative efficacy and dosing unconfirmed
	Resveratrol	C6 astroglial cells	In Vitro Laboratory Study	Inhibits TNF-α, IL-1β; restores GSH homeostasis	Limited to in vitro data; neurorestorative efficacy and dosing unconfirmed
	Lycopene	Sprague–Dawley rats	In vivo experimental study	Inhibits lipid peroxidation; activates Nrf2/HO-1; suppresses NF-κB signaling and IL-1α/TNF-α; modulates apoptosis (↑Bax, ↓Bcl-2)	Limited to animal models; poor BBB permeability; no long-term safety data
Anti-excitotoxicity	MK-801	Rat brain synaptosomes	In Vitro Laboratory Study	Acts as an NMDA antagonist to block excitotoxicity, reducing calcium influx and oxidative stress; mitigates seizures and rotational behavior psychiatric/cognitive/neurodevelopmental risks; unfavorable pharmacokinetics precludes chronic use	Limited to animal models; psychiatric, cognitive and neurodevelopmental risks
Antiapoptotic	Exosome-based miRNA therapy	Mouse neuronal HT22 cells and Kunming mice	A combined in vitro and in vivo study with human sample validation	Modulates apoptosis (↑Bcl-2/Bax ratio, ↓caspase-3 activation); transmigrates across the BBB to deliver neuroprotective miRNAs (e.g., miR-199a-3p, miR-9), suppressing pathogenic pathways (e.g., MAPK signaling)	Limited to animal models; incomplete cargo characterization; lack of neuron-specific exosome isolation and characterization methods; inter-donor miRNA variability; requires clinical validation

*NA* Not Available, indicating this strategy is theoretically supported but lacks experimental validation in methylmalonic acidemia models (cellular, animal, or clinical)

*Abbreviations*: *Bax* Bcl-2-associated X protein, *Bcl-2* B-cell lymphoma 2, *BHMT* betaine-homocysteine methyltransferase, *CoQ10* coenzyme Q10, *CPS1* carbamoyl phosphate synthetase 1, *CRP* C-reactive protein, *LNP* lipid nanoparticle, *MDA* malondialdehyde, *NCAM-1* neural cell adhesion molecule-1, *PGC-1α* peroxisome proliferator-activated receptor gamma coactivator 1-α, *PPARγ* peroxisome proliferator-activated receptor gamma, *PINK1* PTEN-induced kinase 1

#### Conventional therapies

Current management of MMAemia focuses on reducing peripheral accumulation of toxic metabolites (Ling et al. 2025; Forny et al. 2021). Standard interventions include dietary branched-chain amino acid restriction to limit precursor influx, Cbl supplementation in vitamin B12-responsive patients to augment residual MMUT activity, carnitine administration to promote propionylcarnitine excretion, and betaine supplementation to promote Hcy remethylation. Adjunctive short-term antibiotics may attenuate gut microbiota-derived propionate synthesis. Liver transplantation is reserved for medically refractory cases to reconstitute MMUT function. During metabolic crises, ammonia scavengers (e.g., sodium benzoate, sodium phenylacetate) or NAG analogs (e.g., carglumic acid) enhance ureagenesis by activating carbamoyl phosphate synthetase 1, thereby reducing hyperammonemia (Yap et al. 2024). Adequate caloric intake is essential during acute decompensation to suppress lactic acidosis (Forny et al. 2021). Although current interventions—endorsed by clinical guidelines and considered standard of care—help stabilize systemic metabolism and may modestly improve CNS outcomes by reducing organic acid accumulation, their efficacy in preventing irreversible neuronal damage remains limited. This limitation stems from the fact that organic acids act as triggers for downstream pathological cascades, including the release of reactive oxygen species (ROS) and pro-inflammatory cytokines. Metaphorically, they serve as the "key to Pandora’s box," initiating self-amplifying processes that continue to drive neurodegeneration even after the primary metabolites have been cleared. Therefore, there is a compelling need for combinatorial therapeutic strategies that directly target the core mechanisms of neuronal injury. Many such interventions are currently in preclinical or early clinical development, and require rigorous translational studies to assess their efficacy in the context of MMAemia.

#### Neuroprotective pharmacotherapies

Pharmacological interventions for metabolite-induced CNS injury primarily target four pathological mechanisms: energy depletion, oxidative stress, neuroinflammation, and excitotoxicity. Disodium citrate and dimethyl-oxoglutarate bypasses the MMUT deficiency-induced metabolic block while partially restoring TCA cycle flux (Luciani et al. 2021; Forny et al. 2023). Creatine stabilizes ATP levels via the creatine-phosphate system, while reducing both lactate accumulation and NMDA receptor overactivation (Vasques et al. 2006). Coenzyme Q10 activates MRC complexes II-III and enhances ATP synthesis (Proctor et al. 2020; Liu et al. 2022), while the PPARγ agonist bezafibrate upregulates PGC-1α to stimulate mitochondrial biogenesis, jointly improving MRC functional efficiency (Costa et al. 2023). Collectively, these interventions sustain neuronal bioenergetics by preserving TCA cycle flux, enhancing MRC function, and stabilizing ATP homeostasis, thereby preventing energy depletion. Antioxidants (e.g., ascorbate, α-tocopherol)(Fernandes et al. 2011; Pettenuzzo et al. 2003; Liu et al. 2022) and mitochondria-targeted agents (e.g., Mito-TEMPO, MitoQ) (Luciani et al. 2020) directly scavenge ROS, supplementation with glutathione precursors (e.g., N-acetylcysteine) may further ameliorate oxidative stress (Lu et al. 2025), while costunolide activates PINK1/Parkin-mediated mitophagy to clear damaged mitochondria (Fu et al. 2025). Several pleiotropic compounds, including lycopene, melatonin, resveratrol, aminoguanidine and carnitine, concurrently mitigate oxidative stress and neuroinflammation through pleiotropic actions (Fernandes et al. 2011; Almeida et al. 2022; Li et al. 2017; Erten 2021; Oja et al. 2017). Furthermore, apitoxin, costunolide, and lycopene demonstrate neuroprotection by inhibiting oxidative damage and modulating apoptosis (Erten 2021; Khalil et al. 2015; Fu et al. 2025). Excitotoxicity is mitigated through distinct mechanisms: NMDA receptor blockade (e.g., MK-801) (Fernandes et al. 2011; Rigo et al. 2006); cannabinoid receptor activation (e.g., WIN55,212–2) (Colín-González et al. 2015); and acetylcholinesterase modulation by caffeic acid/caffeine (Portela et al. 2021). Notably, all these compounds exhibit antioxidant properties through different pathways. These multifaceted interventions highlight the potential for combinatorial therapies to address the complex pathophysiology of metabolic CNS injury.

#### Emerging pharmacotherapies

Novel therapeutic strategies aimed at reducing toxic metabolite accumulation are currently under active investigation for MMAemia. HST5040 (2,2-dimethylbutyrate) (Armstrong et al. 2021), a prodrug metabolized to HST5040-CoA, modulates the CoA pool to attenuate propionyl-CoA and methylmalonyl-CoA accumulation. Preclinical studies indicate ancillary benefits, including ammonia clearance, restoration of energy homeostasis, and preservation of mitochondrial integrity. Notably, its Phase II clinical trial (NCT04732429) was discontinued in January 2024, with outcomes yet to be published. While BCAT1/2 inhibition is theoretically promising for MMAemia (reducing branched-chain amino acid-derived toxic metabolites), research shows it fails to lower core disease biomarkers or rescue animal model phenotypes, making it unlikely a standalone effective therapy (Hemmingsen et al. 2025).

#### Gene and exosome therapeutics

Novel therapeutic approaches, including gene therapies and exosome-based delivery, aim to restore MMUT function or mitigate metabolite-induced toxicity. Preclinical studies demonstrate the potential of adeno-associated virus (AAV)-mediated *MMUT* or *SIRT5* gene delivery, the latter modulating methylmalonylation (Dimitrov et al. 2021; Head et al. 2022). Clinical trials (NCT03810690, NCT04899310, NCT05295433) are currently evaluating lipid nanoparticle-formulated *MMUT* mRNA (mRNA-3704, mRNA-3705) to enable hepatic conversion of methylmalonyl-CoA to succinyl-CoA. While gene editing for *MMUT* allele correction shows preclinical promise, key challenges persist in delivery precision, off-target effects, and safety profiling (Coughlan et al. 2024).

Exosome therapy represents a cell-free approach with multiple advantages (Chen et al. 2026). These 30–150 nm lipid bilayer vesicles, secreted by cells and derived from intracellular multivesicular bodies, serve as biomarkers, drug delivery vehicles, and therapeutic targets (Di et al. 2024). In MMAemia, levels of MMA in serum total exosomes and neuron-derived exosomes are significantly elevated, correlating with cognitive impairment severity (Sun et al. 2022). Their native lipid bilayer structure ensures high biocompatibility, low immunogenicity, and efficient BBB penetration. Exosomes readily traverse both the BBB and placental barrier, demonstrating significant therapeutic potential for inborn metabolic diseases through potential fetal-stage diagnostic and neuroprotective applications to prevent neurotoxic metabolite-induced brain damage (Dimitrov et al. 2021; Rehman et al. 2023). Therapeutic neural exosomes can deliver diverse therapeutic cargo (nucleic acids, proteins, lipids, and small-molecule drugs), enabling synergistic multi-targeted interventions (Di et al. 2024; Liu et al. 2025; Rai et al. 2025). Notably, exosomes demonstrate cross-species tolerance (Di et al. 2024). For instance, human plasma-derived exosomes suppress hippocampal neuronal apoptosis and enhance spatial learning and memory in MMAemia mice, primarily through their cargo miRNAs (e.g., miR-9, miR-199a) that regulate apoptosis-related pathways (Zhou et al. 2024). From a clinical translational perspective, exosomes exhibit several advantageous characteristics: (1) exceptional storage stability under optimized conditions (Di et al. 2024), (2) enhanced targeting specificity through genetic modification, chemical conjugation, or nanomaterial functionalization (Chen et al. 2026), and (3) multiple administration routes (intranasal, oral, intravenous) (Rehman et al. 2023; Qiu et al. 2025). However, technical challenges persist, particularly in purification processes (incomplete removal of endogenous procoagulants and vesicle damage) and targeted delivery (compromised by imperfect targeting, causing ectopic accumulation and related adverse effects) (Salimi et al. 2023). Notwithstanding these limitations, exosome-based therapeutics represent a highly promising modality for clinical applications.

#### Precision medicine and combinatorial targeted interventions

Future therapeutic innovation in MMAemia should integrate two complementary strategies: (1) precision medicine guided by individual metabolite and biomarker profiles, and (2) combinatorial interventions targeting key pathophysiological pathways, enhanced by delivery systems capable of penetrating the BBB.

The first approach leverages neuron-derived exosomal biomarkers for early risk stratification, enabling personalized therapeutic regimens based on distinct CNS pathogenic signatures. For example, patients with MMAemia-HC who exhibit a pro-inflammatory profile may benefit from combined cobalamin/betaine therapy and NLRP3 inflammasome inhibitors—addressing both homocysteine metabolism and microglial pyroptosis. Conversely, individuals with isolated MMAemia, often marked by severe mitochondrial dysfunction, may respond more favorably to mitochondrial-supportive strategies, such as energy substrate supplementation and mitophagy activation using compounds like costunolide.

The second strategy emphasizes the use of advanced delivery platforms—including engineered exosomes and lipid nanoparticles—to improve CNS drug targeting. Exosomes can be loaded with mitochondria-targeted antioxidants such as Mito-TEMPO, enabling direct neutralization of reactive oxygen species within neurons. Similarly, lipid nanoparticles, already in clinical trials for MMUT mRNA delivery, could be adapted for co-delivery of antioxidants, anti-inflammatory agents, or post-translational modification (PTM) modulators, thereby supporting multi-targeted therapeutic approaches.

By advancing toward personalized, mechanism-specific therapies, these strategies represent a critical evolution beyond conventional metabolic correction—offering new potential to directly mitigate the complex neuropathology of MMAemia.

The therapeutic approaches outlined in this section, which span conventional metabolic control, neuroprotective pharmacotherapies, emerging small molecules, gene/exosome-based interventions, and precision medicine, target discrete steps in MMAemia-driven metabolic and cellular injury cascades. Yet, notable limitations persist for each modality (see Table 2). Given the heterogeneous pathogenesis of MMAemia-associated CNS injury, combinatorial strategies integrating enzyme restoration, metabolite clearance, mechanism-based neuroprotection, and BBB-penetrant delivery hold considerable promise for mitigating irreversible neurological damage.

### Research gaps and future directions in MMAemia

Despite advances in understanding the pathophysiology of MMAemia and emerging therapies, several key gaps hinder clinical translation. First, the complexity of metabolite accumulation remains incompletely characterized. Quantitative and temporal data on metabolite interactions are limited, and it is unclear whether, for example, MMA contributes to acute neuronal injury while homocysteine promotes chronic neurodevelopmental impairment. Longitudinal studies integrating multi-omics, neuroimaging, and functional assessments are needed. Second, the role of aberrant PTMs remains poorly defined. The protein targets and functional consequences of these modifications—particularly their impact on metabolic enzymes and their interaction with metabolite toxicity—are not yet understood. Tandem mass spectrometry coupled with proteomics and functional validation will be essential to address this gap. Third, therapeutic limitations persist. Conventional treatments do not effectively eliminate intracerebral metabolites, and novel approaches face barriers such as blood–brain barrier penetration and immunogenicity. Future research should prioritize translational studies to validate combinatorial, mechanism-targeted strategies for addressing the multisystemic pathology of MMAemia.

## Conclusions

MMAemia is a complex metabolic disorder in which toxic metabolites such as MMA, 2-MCA, PA, Hcy, ammonia, and lactate contribute to brain injury through mitochondrial dysfunction, neuroinflammation, and excitotoxicity. These mechanisms act synergistically, disrupting energy metabolism, redox homeostasis, glial function, and synaptic signaling.

While current therapies help manage systemic metabolism, they fail to address the ongoing accumulation of neurotoxic metabolites in the brain. This highlights the urgent need for CNS-targeted interventions that modulate mitochondrial function, suppress neuroinflammation, and restore neurotransmitter balance. Emerging evidence also implicates abnormal PTMs (e.g., methylmalonylation and lactylation) as critical contributors to metabolic stress and neuroinflammatory signaling, warranting further investigation.

Future efforts should focus on characterizing the temporal progression of damage, elucidating synergistic metabolite interactions, identifying novel core therapeutic targets within dysregulated pathways, and develop personalized strategies to counteract MMAemia-induced neurodevelopmental impairments through multifactorial interventions. Addressing metabolite-driven neurotoxicity holds promise not only for MMAemia but for other neurometabolic and neurodegenerative conditions as well.

## Data Availability

Not applicable.

## Abbreviations

AAV: Adeno-associated virus;
AdoCbl: Adenosylcobalamin;
BBB: Blood–brain barrier;
CAT: Catalase;
Cbl: Cobalamin;
CNS: Central nervous system;
ECM: Extracellular matrix;
GABA: γ-Aminobutyric acid;
GAD: Glutamate decarboxylase;
GDH: Glutamate dehydrogenase;
GPx: Glutathione peroxidase;
GS: Glutamine synthetase;
GSH: Glutathione;
Hcy: Homocysteine;
IL: Interleukin;
iNOS: Inducible nitric oxide synthase;
KGDHC: α-Ketoglutarate dehydrogenase complex;
LDH: Lactate dehydrogenase;
MeCbl: Methylcobalamin;
MMA: Methylmalonic acid;
MMAemia: Methylmalonic acidemia;
MMUT: Methylmalonyl-CoA mutase;
MRC: Mitochondrial respiratory chain;
NAG: N-acetylglutamate;
NAGS: N-acetylglutamate synthase;
NF-κB: Nuclear factor κB
NMDA: N-methyl-D-aspartate;
NO: Nitric oxide;
NOS: Nitric oxide synthase;
OXPHOS: Oxidative phosphorylation;
PA: Propionic acid;
PDH: Pyruvate dehydrogenase;
PTM: Post-translational modification;
RNS: Reactive nitrogen species;
ROS: Reactive oxygen species;
SDH: Succinate dehydrogenase;
SOD: Superoxide dismutase;
TCA: Tricarboxylic acid (cycle);
TNF-α: Tumor necrosis factor-α;
2-MCA: 2-Methylcitric acid
